# REDUCING AVOIDABLE FACILITY TRANSFERS: THE RAFT MODEL

**DOI:** 10.1093/geroni/igz038.2829

**Published:** 2019-11-08

**Authors:** Daniel Stadler

**Affiliations:** Dartmouth-Hitchcock Medical Center, Lebanon, New Hampshire, United States

## Abstract

Reducing Avoidable Facility Transfers (RAFT) is a Dartmouth-developed program that identifies and honors “what matters most” to patients residing in skilled nursing facilities in a value-based, sustainable way. RAFT aims to reduce avoidable facility transfers of older adults from long-term care and post-acute care facilities to emergency departments (ED). Key components of RAFT presently include (1) systematically eliciting goals of care for all skilled nursing facility residents, (2) translating these goals into orders using the Physician Orders for Life-Sustaining Treatment form, (3) documenting patient wishes about hospitalization, and (4) ensuring that these wishes inform decision-making during acute crises. Data from a pilot program, begun in 2016 with three rural skilled nursing facilities in collaboration with the Dartmouth-Hitchcock Medical Center geriatric practice, showed a 35% reduction in monthly ED transfers, a 30.5% reduction in monthly hospitalizations, and a 50.7% reduction in monthly ED and hospitalization-related charges.

